# Modulation in phase and frequency of neural oscillations during epileptiform activity induced by neonatal Zika virus infection in mice

**DOI:** 10.1038/s41598-020-63685-2

**Published:** 2020-04-21

**Authors:** Daniel J. L. L. Pinheiro, Leandro F. Oliveira, Isis N. O. Souza, João A. Ferres Brogin, Douglas D. Bueno, Iranaia Assunção Miranda, Andrea T. Da Poian, Sergio T. Ferreira, Claudia P. Figueiredo, Julia R. Clarke, Esper A. Cavalheiro, Jean Faber

**Affiliations:** 1 Department of Neurology and Neurosurgery – Paulista School of Medicine – Federal University of São Paulo (UNIFESP), São Paulo, Brazil; 2 School of Pharmacy – Federal University of Rio de Janeiro (UFRJ), Rio de Janeiro, RJ 21944-590 Brazil; 3 Department of Mechanical Engineering – São Paulo State University, Ilha Solteira, SP 15385-000 Brazil; 4 Department of Mathematics – São Paulo State University, Ilha Solteira, SP 15385-000 Brazil; 5 Institute of Microbiology Paulo de Goes, Federal University of Rio de Janeiro (UFRJ), Rio de Janeiro, RJ 21944-590 Brazil; 6 Institute of Medical Biochemistry Leopoldo de Meis, Federal University of Rio de Janeiro (UFRJ), Rio de Janeiro, RJ 21944-590 Brazil; 7 Institute of Biophysics Carlos Chagas Filho, Federal University of Rio de Janeiro (UFRJ), Rio de Janeiro, RJ 21944-590 Brazil; 8 Nucleus of Neuroengineering and Computation – Institute of Science and Technology – Federal University of São Paulo (UNIFESP), São Paulo, Brazil

**Keywords:** Dynamical systems, Epilepsy

## Abstract

Modulation of brain activity is one of the main mechanisms capable of demonstrating the synchronization dynamics of neural oscillations. In epilepsy, modulation is a key concept since seizures essentially result from neural hypersynchronization and hyperexcitability. In this study, we have introduced a time-dependent index based on the Kullback-Leibler divergence to quantify the effects of phase and frequency modulations of neural oscillations in neonatal mice exhibiting epileptiform activity induced by Zika virus (ZIKV) infection. Through this index, we demonstrate that fast oscillations (gamma and beta 2) are the more susceptible modulated rhythms in terms of phase, during seizures, whereas slow waves (delta and theta) mainly undergo changes in frequency. The index also allowed detection of specific patterns associated with the interdependent modulation of phase and frequency in neural activity. Furthermore, by comparing ZIKV modulations with the general computational model *Epileptors*, we verify different signatures related to the brain rhythms modulation in phase and frequency. These findings instigate new studies on the effects of ZIKV infection on neuronal networks from electrophysiological activities, and how different mechanisms can trigger epilepsy.

## Introduction

Zika virus (ZIKV) is an arbovirus from the *Flaviviridae* family, which was first reported in 1947 in Uganda^1^. It is mainly transmitted by the *Aedes aegypti* mosquitoes, but can also be transmitted sexually and by blood transfusion from an infected donor^2,3^. Zika has been considered as an emergent health threat, given its epidemic history, transmission in tropical areas^4^, neurological, congenital diseases^5,6^, as well as given the fact that it is associated with brain abnormalities in newborns (Caires-Júnior *et al*.^7^; Rasmussen *et al*.^8^). Recent reports show that epileptic seizures are among the main neurological outcomes of congenital Zika syndrome (CZS)^9–13^; additionally, reports of the incidence of epileptic seizures in infants exposed to the ZIKV and who had not developed microcephaly^14^, represent new challenges due to changes in the neurodevelopmental stages and even their long-term consequences.

Epilepsy is one of the most common neurological disorders worldwide^15^, clinically characterized by the occurrence of at least two unprovoked seizures in less than 24 hours, high unprovoked seizure recurrence risk, or even by the diagnosis of epilepsy syndrome^16^. Despite important advances in the understanding of the involved pathophysiology, multiple mechanisms behind the hyperexcitability and hypersynchronization of neurons during epileptic seizures, still need to be understood better. For instance, the role of neuronal discharge modulation during seizures and their relationship with epileptogenesis is not yet completely elucidated^17^. A physical approach describing the neuromodulatory mechanisms during seizures can provide new insight into the commonly investigated electrophysiological features^18–22^.

Neural oscillations can be described in terms of extracellular potentials, called local field potentials (LFP)^23^, which are mainly characterized by their amplitude, frequency, and phase. LFP modulation is directly associated with the synchronization of the neuronal inputs, and how the neural populations are structurally and functionally organized^23–25^. Synchronization occurs essentially through changes of the oscillatory rhythms in specific frequencies, decreasing the phase differences among neural oscillations. This interdependence between phase and frequency can be described in terms of a modulatory process that control the information flow within neuronal populations and brain regions^23–25^. In this way, the strength of phase-frequency coupling is directly related to the synchronicity of the neural network. That is, strong couplings brings up hypersynchronous dynamics among the network unities^26^.

Since electrophysiological neural activities are a physical oscillatory phenomenon, they exhibit interactive dynamics given by different types and levels of synchronous effects^26^. If the synchronous dynamics of a neuronal population is related to its phase and frequency interactions, by identifying specific rhythms responsible to coordinate the whole activity during seizures can help to elucidate and characterize underlying mechanisms of epilepsy, such as hypersynchronicity during seizures promoted by different agents. Specifically, researches have been reporting that the fast oscillations work as a facilitator for synchronization process, in a long-range communication between neuronal groups^27,28^. Therefore, the understanding of how fast and slow oscillations modulate seizures is a critical point for a better description of epileptogenic process.

We investigated the regime transitions of neural oscillations in neonate mice infected with ZIKV that presented recurrent epileptic seizures. Mainly, we focused on the phase and frequency changes, here identified as a modulatory effect on the neural oscillations. We adapted the Kullback-Leibler divergence (D_KL_)^29^ to explore and quantify the effect of phase and frequency interdependencies on epileptiform activities. Our results show that fast oscillations, namely gamma and beta 2 waves, are predominantly modulated in brain activity during seizures. Since ZIKV infection causes an imbalance in brain rhythms, the results raise the question whether this phase and frequency modulatory mechanism is promoted specifically by ZIKV or whether it is a general characteristic of epileptiform activities.

## Results

### Power frequency characterization

**Figure 1:**
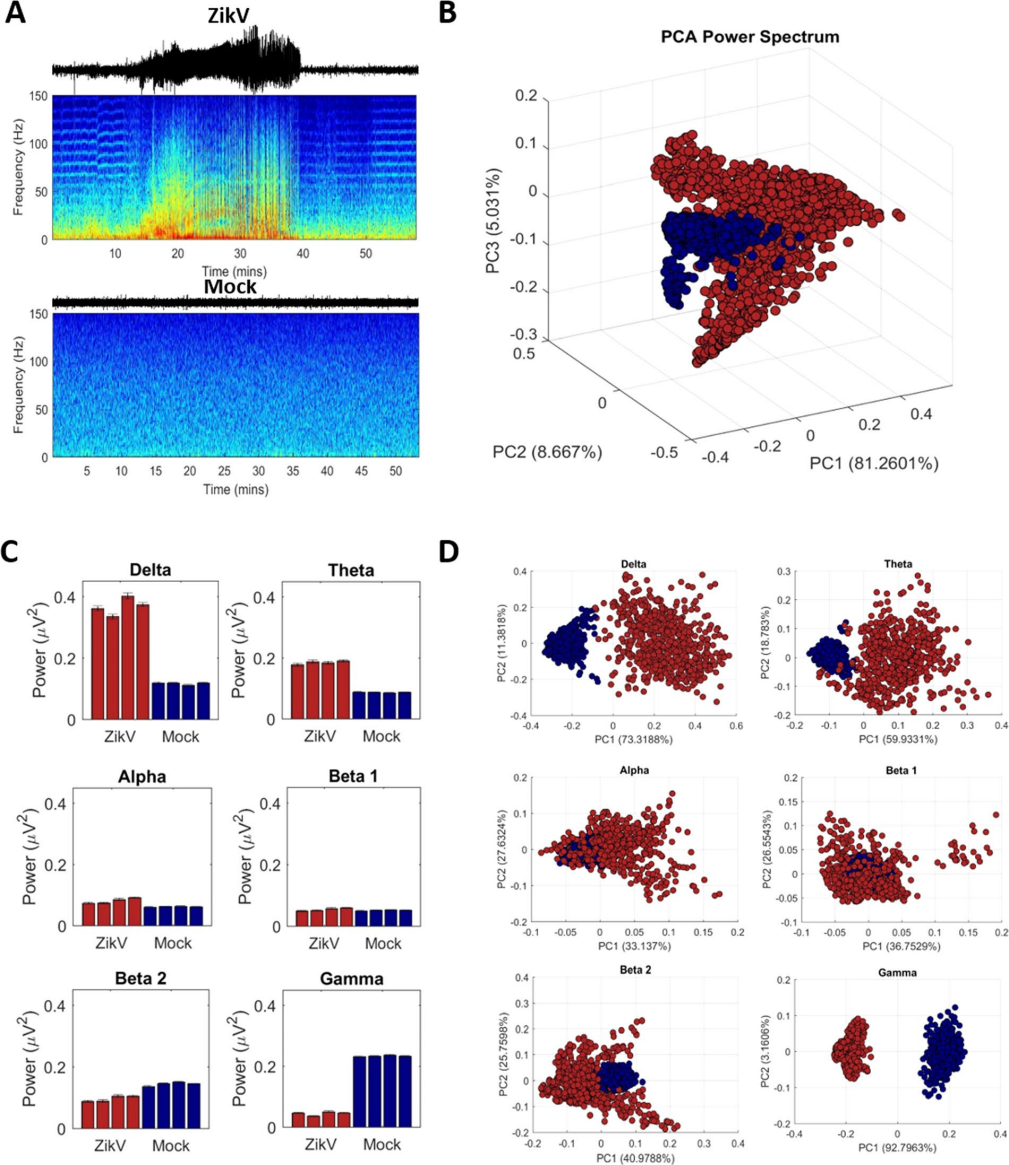
Frequency analysis of electrophysiological signal from neural activity of neonate mice infected with Zika Virus (ZikV) in comparison with control group (Mock). (**A**) Example of spectrogram from each animal group, notice that the power fluctuation between 15–40 minutes in ZikV signal is related to epileptic seizure. There are also differences in the frequency bands influences even in preictal, postictal and baseline activity when compared with Mock. (**B)** Principal Component Analysis (PCA) of power spectrum with the percentage of explained variance in each axis. Note that ZikV and Mock has frequency behavior, and that the first one shows a higher variability of some brain rhythms. **(C)** Frequency power bands of neuronal rhythms per animal (representation of mean ± confidence interval for mean 95% of reliability). **(D)** PCA of individual brain rhythms. Stands out that in most of frequency bands is perceptible the clusters referent of each experimental group, mainly in δ and γ bands.

We first analyzed the spectral patterns of recordings from the Mock or ZIKV-infected mice, characterizing their signatures in the frequency, Fig. 1A. Two representative animals were chosen to illustrate the general aspects observed from each group, and all other spectrograms are presented in the supplementary material (Fig. S1). From this figure, it is possible to note that in Mock-infected mice there was no power predominance of any specific frequency band, but ZIKV-infected mice showed seizure events starting around 14 minutes of the record, and even for the baseline activities changes were noted in its spectral signature. Previous work showed that mice infected with ZIKV presented with at least two seizures events during a period of 2 hours, with spiking activity followed by polyspike-waves or fast sharp waves, exhibited through invasive electroencephalography as well as behavioral analysis^30^.

Preictal and postictal epochs, in ZIKV-infected group, also show fluctuations of their PSD, mainly for low-frequency bands. It is noteworthy that at the beginning and at the end of seizures there were consistent increases in power on the high-frequency bands that did not persist along the rest of the signal. This phenomenon has been discussed as a precursor of the epileptiform spike discharges^31^. The increase of fast oscillations, such as gamma, are discussed as precursors in the pre-ictal period but, in fact, there remnants of these changes in the post-ictal period, may occur as the epileptiform activity is ceasing.

Figure 1C shows a direct comparison using the CIs for the means, between the PSD of each mouse of the ZIKV and Mock groups. There is a consistent and significant difference in mean power for all frequency bands between both the groups. The main differences are presented in δ, θ, β_2,_ and γ oscillations. By applying PCA analysis on the PSD, considering all animals (Fig. 1B), without band selection, it was still possible to observe a clustering between both the groups, despite some intersection between them. When PCA is applied onto each specific brain rhythms, Fig. 1D, the clustering formation corroborates the previous mean differences, but now highlighting a self-similarity intragroup and differences intergroup, especially for δ, θ, and γ rhythms. Particularly, γ oscillations exhibit a profile with lower variance intragroup and higher variance intergroup. These observed patterns give lead to the spectral signature of the recordings, but does not explain the modulatory effects that is happening on the phase and frequency of the brain rhythms.

## Modulatory effects using ${{\boldsymbol{\Lambda }}}_{{\boldsymbol{j}}}^{{\boldsymbol{k}}}$ index

**Figure 2:**
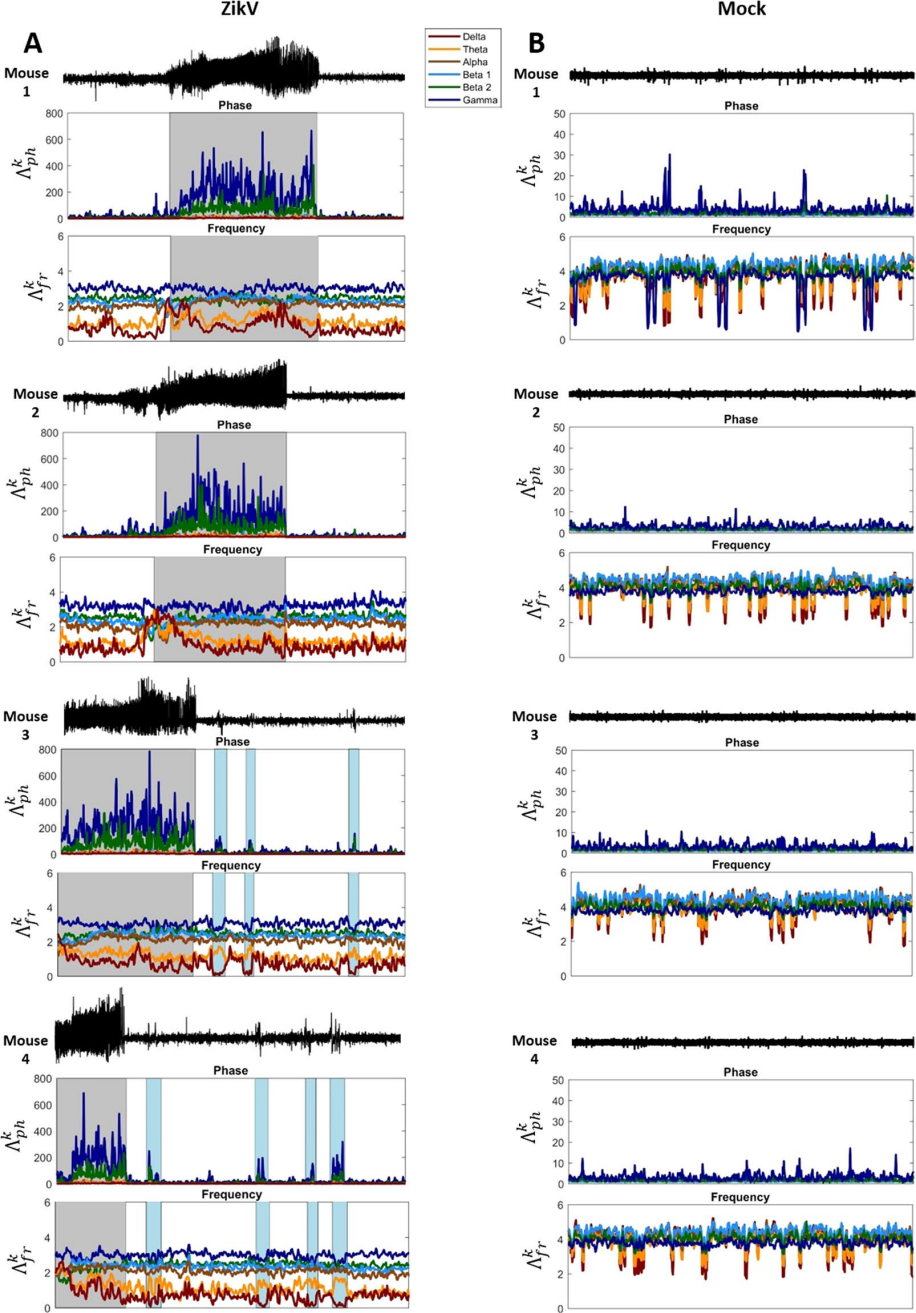
${\Lambda }_{{\rm{j}}}^{k}$ index variation over the time recorded using phase and frequency as features. In **B**, Mock mice, shows that the fast oscillations (β_2_ and γ) are the most modulated rhythms, although, their modulation effects in the frequency power does not gives rise to any rhythms in specific, what is expected considering that there is no specific task during the records. Mice injected with ZIKV (**A**), in other way, shows an already changed pattern wherein the slow oscillations have visually lower influences in the raw signal. Evaluating the ${\Lambda }_{ph}^{k}$ index variation in these animals, notice that the modulation of γ and β_2_ wave are more intense mainly in seizure (gray shadow), and their effects in frequency seems to change δ and θ rhythms. Worth to emphasize that there are some high peaks of modulation in the signal by fast oscillations, the blue shadows when the mouse is not having seizure, that provokes changes in the slow oscillations (δ and θ) and can represent the nature of system that always trends to increase the level of γ and β_2_ modulation.

After general characterization of the signal power spectral signature, the ${\Lambda }_{j}^{k}$ index in frequency and phase was calculated, referred here as ${\Lambda }_{fr}^{k}$ and ${\Lambda }_{ph}^{k}$, respectively. Figure 2 shows how ${\Lambda }_{j}^{k}$ index variates along the entire recordings, for all animals. Plots on the right-hand side display the ${\Lambda }_{fr}^{k}$ and ${\Lambda }_{ph}^{k}$ for ZIKV-infected animals, whereas plots on the left-hand side display for Mock. Considering the ${\,\Lambda }_{ph}^{k}$ patterns, β_2_ and γ oscillations are the predominant modulated phase rhythms for both groups. However, for ZIKV mice, Fig. 2A, the ${\,\Lambda }_{ph}^{k}$ present a considerable increase, with high variation, specifically during the seizure epochs (marked with gray shadows), emphasizing abrupt changes in the modulation process of these rhythms. Additionally, along with the entire ZIKV recordings there are recurrent short periods of sudden increase of ${\,\Lambda }_{ph}^{k}$ for these rhythms, β_2_ and γ, related to brief epileptiform discharge periods (blue shadow in Fig. 2A.2-A.4). These short periods present a lower magnitude compared to the seizure epochs. Considering Mock group, ${\Lambda }_{ph}^{k}$ shows a constant modulation along the whole recording, where the predominance of β_2_ and γ is also higher in comparison to the other rhythms. However, this modulation presents a magnitude level of about 10 un, which is considerably lower in comparison to ZIKV group, especially during seizure epochs.

Considering the frequency, for all *k-*rhythms, ${\Lambda }_{fr}^{k}$ also reveals different influences of each specific rhythm along the LFP recordings. Mock ${\,\Lambda }_{fr}^{k}$ index shows a modulation pattern that are indistinctly, except some moments of higher variation of δ and θ. In contrast, ZIKV ${\Lambda }_{fr}^{k}$ index shows a totally different modulation of each frequency in the LFP recordings, especially during seizure epochs. ZIKV ${\Lambda }_{fr}^{k}$ shows that δ and θ are the most affected in this condition, having a specific modulated pattern with sudden high peaks of ${\Lambda }_{ph}^{k}$ in β_2_ and γ bands.

**Figure 3:**
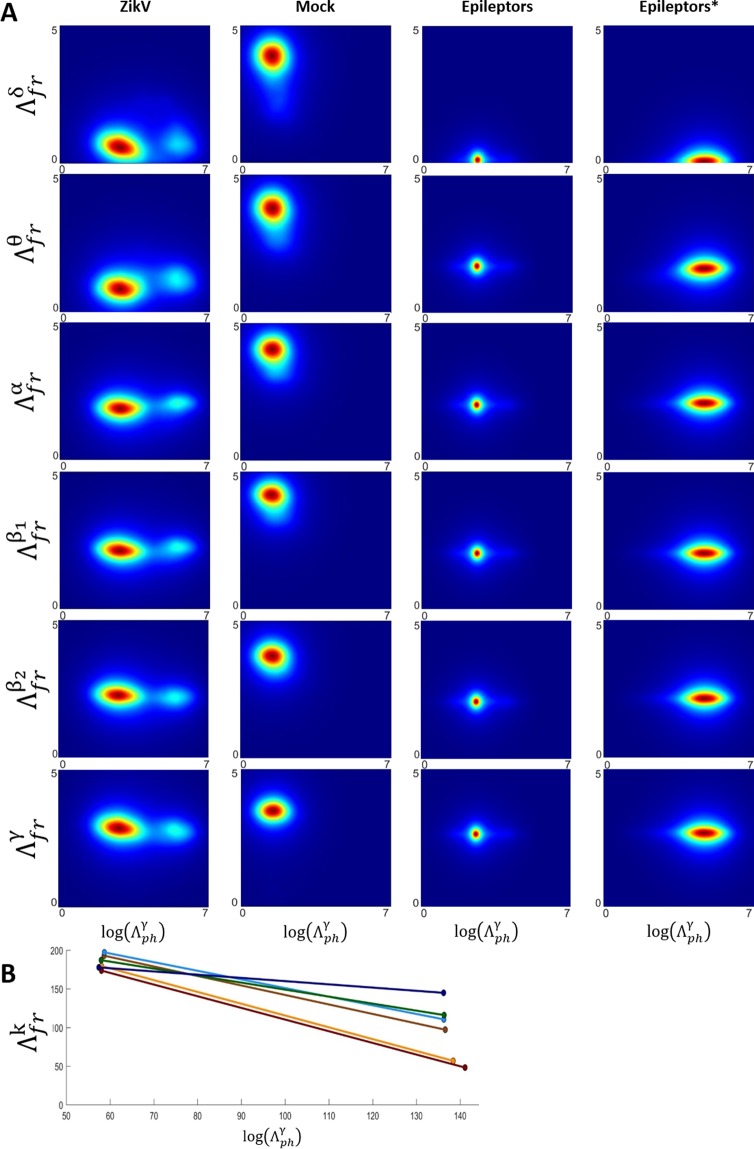
Density plot of the relationship between modulation on phase γ band and modulation on all frequency brain rhythms. (**A**) The ${\Lambda }_{ph}^{{\rm{\gamma }}}$ axis shows the most modulated oscillation in function of ${\Lambda }_{fr}^{{\rm{k}}}$, ∀ *k-*rhythms. The first and second column represent the association between phase and frequency for ZIKV and Mock animals, highlighting the differences associated with each modulatory response. The columns three and four exhibit two different epileptiform-like patterns (‘*’ refers to a modification of parameters used in the simulation, as explained in Supplementary methods), which compared to ZIKV patterns emphasize the high sensitivity of $\,{\Lambda }_{j}^{k}$ to detect intrinsic features associated with each activity. Figure B shows the centroids of each density plot of A, referent to ZIKV and Mock mice, highlighting the discrepancy between the modulation level of γ in phase in relation to other rhythms inn frequency.

Figure 3 exhibit a density plot graphics of the ${\Lambda }_{j}^{k}$ index, fixing *ph* = γ against all other *k-*rhythms for frequency. The ${\,\Lambda }_{ph}^{k}$ was fixed in γ since it presented the most prominent modulatory effect during the seizures. This analysis shows how the modulatory effects in the phase are related to the modulatory effects observed in the frequency. These graphics could help to evaluate if different physiological conditions would present different modulatory effects between both phase and frequency. Although these plots do not imply phase-frequency coupling, they depict how the most modulated phase, ${\Lambda }_{ph}^{{\rm{\gamma }}}$, exhibits an association with all rhythms, in the frequency, but with different aspects, Fig. 3. The β_2_ rhythm also presents the same profile as γ, with a lower level of modulation along the whole signal. It is important to notice in these density plots the differences between data from ZIKV and Mock mice, which already exhibit specific configurations associated with the modulatory effects of phase and frequency for all *k*-rhythms. Concerning that we are not able to affirm if the origins of these electrophysiological patterns come from ZIKV infection or from a common epileptic effect, since we did not perform any experiment with neonate mice with epilepsy without ZIKV-infection, we decide to use computational models of epilepsy to see if they are general patterns. Although, in order to instigate better this discussion, we added a new comparison using simulated electrophysiological patterns yielded by a computational model of epilepsy, known as Epileptors^32^ – Supplementary methods. In this way, we were able to highlight the sensibility of the index considering its accuracy to distinguish more types of epileptiform activities.

**Figure 4:**
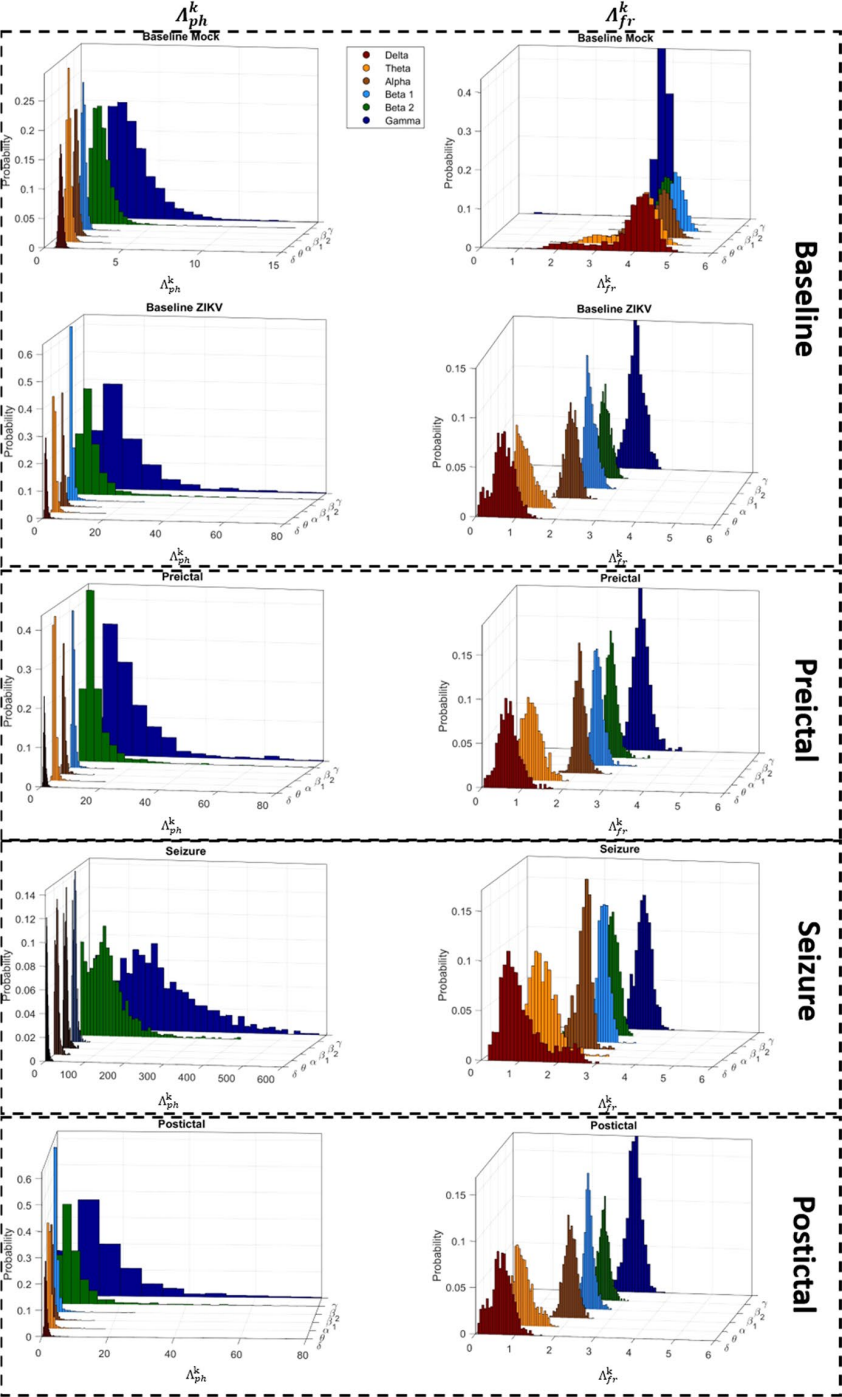
Histograms of ${\Lambda }_{ph}^{k}$ and ${\Lambda }_{fr}^{k}$ indexes for each stage of recordings. In ${\Lambda }_{ph}^{k}$ it is possible to note that the modulatory effects of all brain rhythms in the baseline, preictal and postictal stages of ZIKV mice has some difference in comparison to baseline Mock, which can be emphasize by the magnitude scale of them that goes up to 80 un of ${\Lambda }_{ph}^{k}$ index in front of 15un. In addition, seizure shows a changed of histogram shape to β_2_ and γ wave, which are the most modulated in epileptiform activity. On the other side, in ${\Lambda }_{fr}^{k}$ the parcel of modulation exhibited by each rhythm in baseline of Mock are very equilibrated, which are changed in all stages ZIKV mice. Particularly, in seizures noted that the shape of δ and θ waves are the most who suffers alteration.

To emphasize the statistical differences of the index ${\Lambda }_{j}^{k}$ on neuronal oscillations the histograms associated with each rhythm were produced, for phase and frequency, during the pre-defined four stages of LFP recordings, Fig. 4. Since, the Mock group had no epileptiform activities it was possible to describe it only during the baseline stage. First part of Fig. 4 shows how the indices ${\,\Lambda }_{j}^{k}$ vary during the baseline activity. In the phase, it is possible to see that, although both present high modulatory effects on γ and β_2_ rhythms, the magnitude of ${\,\Lambda }_{j}^{k}$ in the baseline ZIKV group is considerably higher than the Mock group. However, in case of frequency, it is possible to see that each rhythm is modulated in a completely different way for ZIKV, while Mock group presents a regular and homogeneous modulatory effect on all rhythms. In the second part, Fig. 4, show how the indices ${\Lambda }_{j}^{k}$ of ZIKV group variates, during the preictal stage. Due to the difficulty in defining a line between the baseline and preictal activity, both stages present very similar distributions of the modulatory effects on each rhythm.

The third part of Fig. 4 show how the indices ${\,\Lambda }_{j}^{k}$ of ZIKV group varies, during the seizure. In phase, it is possible to see that ${\Lambda }_{ph}^{k}$ exhibits high modulation on γ and β_2_ rhythms, expressed by their scales and dispersions comparatively to all other rhythms. In case of frequency, all ${\Lambda }_{fr}^{k}$ rhythm distributions are very similar to the preictal stage, except δ and θ rhythm that present a considerable modulation on their mean magnitude and dispersion. The fourth part of Fig. 4 show how ${\Lambda }_{j}^{k}$ index varies during the postictal stage, and in both phase and frequency the distributions of each rhythm return to the previous baseline and preictal patterns.

**Figure 5:**
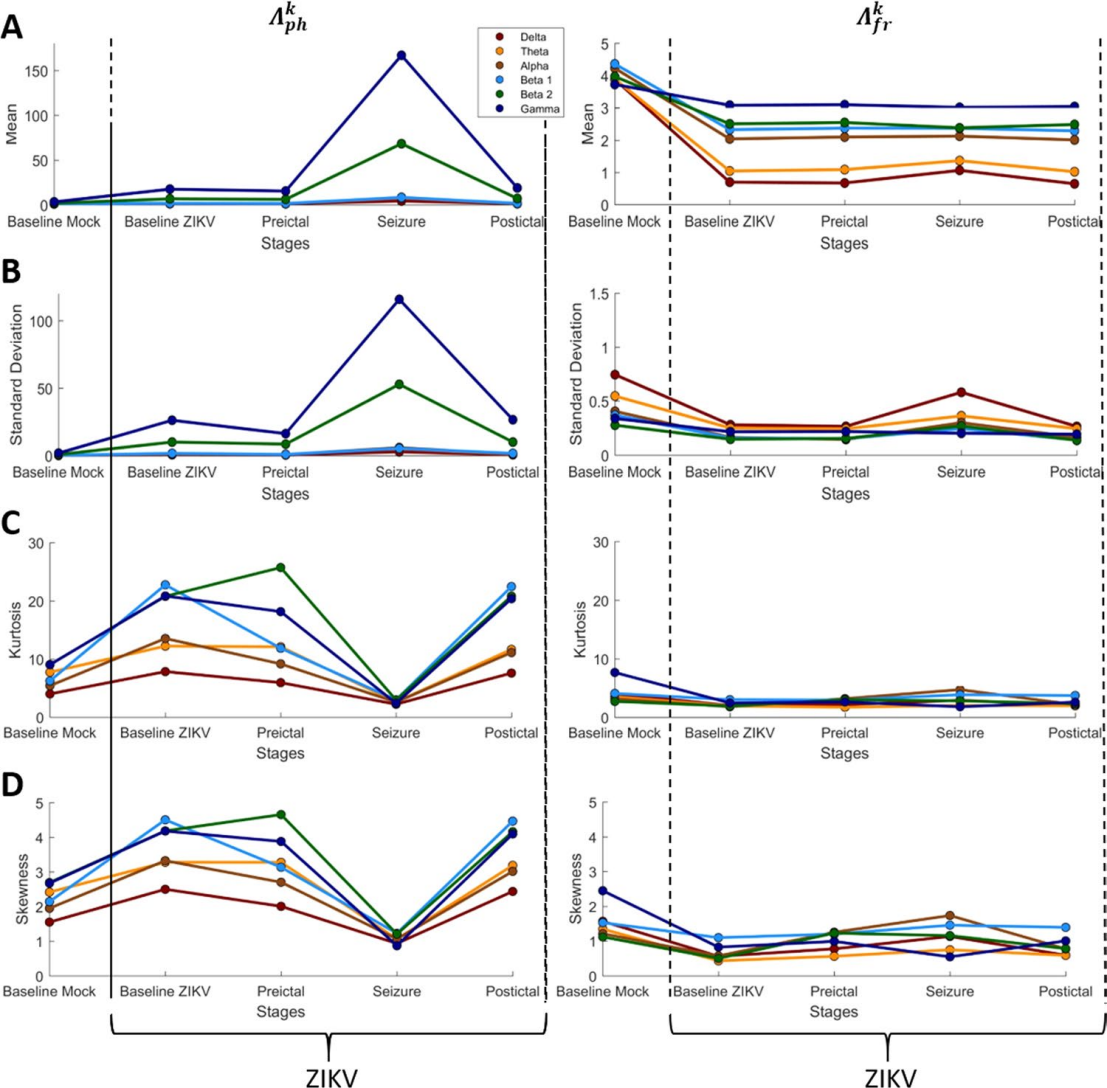
Histograms measures of each stage with ${\Lambda }_{ph}^{k}$ and ${\Lambda }_{fr}^{k}$. Notice that in all measurements the ${\Lambda }_{j}^{k}$ index show an expressive change at seizure stage for all statistical moments, although just the kurtosis and skewness can show these differences in all brain rhythms in phase. Worth to emphasize that in seizure each brain rhythms have more stable modulation level, in other words, the phase of these wave seems to follow a more restrict regime, what can indicate a configuration more appropriated to hyper-synchronization.

To quantify the differences exhibited in the histograms associated to the index ${\Lambda }_{j}^{k}$, the four statistical moments for each one of the stages was calculated and compared, considering each rhythm. Figure 5 emphasizes how the statistical moments (mean, standard deviation, kurtosis, and skewness) of ${\,\Lambda }_{j}^{k}$ varies among the four stages, including the baseline of Mock, for each histogram exhibited previously in Fig. 4. This calculus quantifies the trends of the distributions and the relationship between centrality and dispersion, indicating critical points of the index ${\,\Lambda }_{j}^{k}$. For both the phase and frequency, the index ${\,\Lambda }_{j}^{k}$ did not present significant changes on mean and standard deviation among the stages, except during the seizure stage which presented a high level of modulation for γ and β_2_ for both statistical moments, but only for phase.

In case of phase, Mock ${\Lambda }_{ph}^{k}$ shows lower kurtosis and skewness for all brain rhythm distributions, whilst in the frequency, ${\Lambda }_{fr}^{k}$ indicate that their values have a higher probability to occur around the mean. During the seizure stages, kurtosis and skewness of ${\Lambda }_{ph}^{k}$ present a deflected point indicating that all brain rhythms assume a stable configuration around the mean, where the level of modulations are more symmetrical.

## Discussion

In this study, we have introduced a new perspective on neural oscillations by investigating the effects of modulation of phase and frequency from *in vivo* local field recordings from neonate mice infected with ZIKV and Mock-infected mice. By using the Kullback-Leibler divergence, we proposed a new index, ${\Lambda }_{j}^{k}$, to emphasize these modulatory effects for both phase and frequency. The modulation of a physical signal occurs when one of their main properties (phase, frequency or amplitude) changes in a controlled manner in order to transmit a specific information^33,34^. With this index, we were able to show that the modulatory effects occur basically by statistical temporal transitions from a uniform-like probability distribution to some structured probability distribution of the correspondent feature (phase or frequency). Since, any sudden and consistent changes in statistical pattern of a stochastic variable corresponds to a modulatory effect^35^, through this index, we were able to identify critical points corresponding to the onset of modulation for each stage.

The modulation term used here is a general concept because it comes from the physical nature of interactive oscillator systems, which can be directly associated with neural activities^19^. The neuronal communication is more effective when a message send by neuron population reaches their destiny in their most excitable phase, in other words, the moment where a neuron has more probability to generate an impulse due to an income from an excitatory synapse^24,25,36^. Thus, the concept of synchronization in neural systems can be seen as a natural phenomenon in which different populations of neurons interact with themselves and produce a complex dynamic due to this interaction^37,38^. Therefore, the use of phase and frequency information of neuronal oscillations is one way to understand better the epileptiform activity and their manifestation. Nowadays, the characterization of epileptic seizures uses a more descriptive analysis of wave shape in time, and power of specific frequencies bands^39–41^.

In general, studies seeking modulatory effects on brain rhythms, in different conditions such as epilepsy, aim to identify the factor couplings between signal features of neuronal oscillations, for example, cross-frequency couplings^38,42–44^. In this work, however, the idea is to evaluate changes in the phase and frequency related to each brain rhythm separately, along the time, considering the effect observed in the raw signal. By this analysis, it was possible to have a more general overview associated with global neural network changes promoted by phase and frequency modulations from local neural populations.

From the results, it was possible to show that fast oscillations, β_2_ and γ, are the most modulated rhythms in the phase, especially during epileptiform activity. Although it does not imply necessarily that they are the cause of these activities, it suggests that their high level of modulation (given by the index ${\Lambda }_{ph}^{k}$) is directly related to the seizures. For the Mock group, the modulation levels during fast oscillations, in the phase, was also higher than the slow oscillations but with lower magnitude compared to the ZIKV group, even out of the epileptiform activity.

Several works have investigated the presence of β and γ oscillation in epileptic seizures, finding higher power activities on these bands during seizures^45–48^. In general, γ waves (30–60 Hz) are associated with memory, sensory, and voluntary movement processing^21,49–52^ and for the bottom-up transmissions^53^. While, β oscillations (12–30 Hz) are associated with sensory-motor processing^54^, to maintain normal brain activity^55^, and for top-down transmissions. Some researchers propose patterns of cross-frequency coupling between brain rhythms as electrophysiological biomarkers for neurological disorders, as well as associating them with some specific behavioral or cognitive functions^37,56^. Although some of these patterns have been described as *signal couplings*, using phase, frequency, and amplitude features, they do not attempt to evaluate which specific brain rhythm is modulated during specific electrophysiological activities.

Considering the phase analyzes, the index ${\Lambda }_{ph}^{k}$ can be seen as an alternative metric to quantify transient regimes related to specific brain rhythms. In this way, using ${\Lambda }_{ph}^{k}$ it is possible to analyze if a k-oscillation allows for interaction with other rhythms, by comparing the temporal changes of its empirical probability distributions^26^. The modulatory effects measured in the phase for β_2_ and γ implies that they are more prone to synchronize with specific oscillations. As they participate in top-down or bottom-up processes, they have a structured probability distribution that enables synchronization with specific rhythms. Most probably this *a priori* selectivity in specific sub-range oscillations is the necessary condition to maintain the stability of neural communications^49,53^. If at some point this selectivity is lost, then all phase regimes are more equiprobable, statistically characterized by a uniform-like distribution. During this state of phase uniformity, the synchronization with different rhythms is facilitated, since none phase state is privileged or coupled.

Although the results show that β_2_ and γ are the rhythms prevalently modulated in the recordings even for the Mock group, during epileptiform activities in the ZIKV group, the modulation levels are considerably higher. We can interpret these differences as an effect promoted by the epilepsy. However, since here, the epilepsy is essentially initiated by ZIKV, it suggests that some structural or functional changes in the network arrangements responsible for these oscillations are possible.

Focusing on the ${\,\Lambda }_{fr}^{k}$ index in the frequency, it indicates how much the power of certain rhythm contributes to the composition of raw signal, since it compares the empirical probability distribution made from PSD of the *k*-rhythm with the raw signal. The modulatory effects are evaluated by the ${\Lambda }_{fr}^{k}$ variation over time^50,57^. The results from ${\Lambda }_{fr}^{k}$ show that all brain rhythms have different contributions in the generation of the raw signal in ZIKV mice compared to the Mock group, Fig. 2. However, in ZIKV during epileptiform activity, δ and θ oscillations showed sudden increases associated with β_2_ and γ modulations, described in the phase by ${\Lambda }_{ph}^{k}$ (blue shadows of Fig. 2). During these periods ${\Lambda }_{ph}^{k}$ decreases in magnitude whilst ${\,\Lambda }_{fr}^{{\rm{\theta }}}$ increases, so the modulation peaks of ${\,\Lambda }_{ph}^{{{\rm{\beta }}}_{2}}$ and ${\Lambda }_{ph}^{{\rm{\gamma }}}$ impact particularly in the frequency power of these oscillations. Since phase modulation corresponds to synchronization and desynchronization effects, it suggests that frequency modulatory effects are likely to be promoted by phase modulation.

All the mice were in exploratory activity, without any specific task during the signal acquisition, which imposed a limit to correlate the signal patterns with any possible behavior or cognitive function. Meanwhile, during the seizure epochs all mice exhibited a freezing behavioral pattern, stopping their exploration and showing tail erection^30^. Indeed, changes in the ${\,\Lambda }_{fr}^{k}$ may indicate that a specific brain rhythm suffered power alteration due to activity from a neuronal network or as a result of the interaction among neuronal populations with coherent activity, since δ and θ were related to ${\Lambda }_{ph}^{{{\rm{\beta }}}_{2}}$ and ${\Lambda }_{ph}^{{\rm{\gamma }}}$. Thus, in the case of epileptiform activity it is interesting to study the effects of ${\,\Lambda }_{fr}^{k}$ to explore the correlations between the modulatory rhythms and the behavioral and cognitive manifestations observed during this activity.

Although all the signals in this study were recorded from neonate mice that developed epileptiform activities, the nature of the observed phenomenon could be characteristic of epilepsy having a different etiopathogenesis. Through the signatures observed in the Fig. 3 it is possible to see that different types of epileptiform activity exhibit specific patterns associated with the modulatory effects on phase and frequency.

Despite it seems there is a general signature directly related to the epilepsy phenomenon, the index ${\Lambda }_{j}^{k}$ were able to detect intrinsic features of epileptiform activities yielded from three different mechanisms, ZIKV, Epileptor and Epileptor*. Therefore, the specific patterns found (rhythms most affected by the modulation – Fig. 2; modulation pattern in relation to the phase and frequency of two rhythms – Fig. 3) can be studied as electrophysiological biomarkers related to the epileptogenesis, and can help understand better their implications and dynamics on neuronal network in that condition.

Additionally, despite a more rigorous conduction of new experiments is necessary, some conjectures can be made based on our results and the current literature for ZIKV infection. As β_2_ and γ have higher modulation level, it suggests that the neuronal population activity or the mechanisms responsible to their generation are somehow changed. Since the outbreak of ZIKV infection in Brazil in 2015, there were evidences of abnormal neuronal migration associated with ZIKV^58^. When it comes to neurodevelopmental stages, the neurotransmitters glutamate and GABA play an important role in the neuronal migration. Glutamate, in particular, is responsible to control radial migration of pyramidal neurons and acts in NMDA receptors regulating inhibitory interneurons tangential migration^59^. Both inhibitory interneurons and glutamate neurons are required, with a high level of precision, for synaptic transmission of γ wave oscillations^52^. Therefore, the migration abnormalities could produce changes in the neuronal network arrangements yielding new configuration which leads to changes in the modulation of neural oscillations.

Therefore, we can conjecture that the infection promoted by ZIKV interferes with the structural or functional organization of the neuronal networks, specifically interneurons responsible for fast oscillations. These networks must be affected allowing unbalanced activities, such as epileptiform patterns^60^. However, this interpretation is not enough to explain the origin of the modulatory effects.

Figure 5D emphasizes a characteristic of skewness and kurtosis in the system’s dynamics, the increase in all brain rhythms after the seizure indicates that the system approached a critical transition^61^. During seizure events, the neural system is passes through a transitionary state, which involves all brain rhythm, which somehow changes the interactions between neuronal population producing modulatory dynamics as seen. Besides indicating that the seizure is a point of criticality for the neuronal system, it should be noted that all brain rhythms, although they do not present changes in the average level of modulation, all present changes in these two statistical moments when it comes to the phase. Thus, the modulation of the phase of brain rhythms becomes even more important for analyses of neural communication, and in cases of hypersynchronization.

These changes in the interactions between neuronal population can also be observed in the density plots of Fig. 3, wherein, for all brain rhythms it is observed that the relation between the modulatory effects on frequency versus phase of γ are totally different in ZIKV mice when compared to Mock.

The description of how phase and frequency of neuronal oscillations change with time offers a new perspective on the general physical mechanisms that allow and sustain epileptic seizures. This novel approach offers a new avenue for the traditional characterization using only amplitude shape in time, and power variation for specific frequencies bands^39–41^. Since all the electrophysiological patterns described here are inherently associated with functional arrangements of neuronal networks, to describe modulatory effects is an indirect way to evaluate disruptions on functional network topologies and their working dynamics^62,63^.

Finally, the ${\Lambda }_{j}^{k}$ index proposed in this work can measure modulatory effects related to the dynamics of brain synchronization, which is an important process for neural communication and new studies and experiments to elucidate the electrophysiological signatures in different contexts can be conducted, such as epilepsy and other comorbidities, associated with the underlying mechanisms related to these modulatory effects.

## Methods

### Experimental model and electrophysiological records

All procedures of this study followed the “Principles of Laboratory Animal Care” (US National Institutes of Health) and were approved by the Institutional Animal Care and Use Committee of the Federal University of Rio de Janeiro (protocol #052/2017). ZIKV was isolated from a febrile patient in the state of Pernambuco, Brazil (gene bank ref. number KX197192). The stocks used in the experiments were produced and tittered as previously described in Coelho *et al*.^64^. As described by Souza *et al*.^30^, three-day old Swiss pups were infected subcutaneously (s.c.) with 30 µL of ZIKV (10^6^ PFU) or the same volume of Mock medium (control).

The choice to perform the infection at post-natal day three was because this timeframe of rodent brain development was shown to be comparable to the third trimester of pregnancy in humans^32,65^. Moreover, factors that determine if and how much ZIKV crosses the human placental barrier and reaches the fetus are unknown. In fact, different outcomes have been reported in dizygotic twin pregnancies^7,65,66^. Therefore, by performing neonatal infection we can be sure that all animals are exposed to similar amounts of virus, which is usually not the case for models of vertical transmission. As the control, the same volume of virus-free conditioned medium of C6/36 cells was used (Mock). The virus-free conditioned medium of uninfected C6/36 cells is the most suitable control, since it contains all products of cell metabolism but not the virus. Injections of mock or ZIKV are performed in the same post-natal day, in the same volume and route. All comparisons are made between ZIKV-infected mice and Mock-injected mice.

The method used to the virus infection was developed and characterized as a mouse model of peripheral (subcutaneous) ZIKV infection in immunocompetent neonatal (post-natal day 3) animals by Souza *et al*.^30^. Under these conditions, they found that ZIKV was able to reach and replicate in the brains of neonatal mice causing several behavioral and neuropathological alterations that, in many ways, resemble those of congenital ZIKV syndrome. Moreover, viral replication was accompanied by increased expression of pro-inflammatory mediators in the brain, leading to several behavioral alterations in mice including epileptic seizures^30^.

Nine days post-infection (dpi) pups were chosen randomly to be submitted to a surgical procedure for the implantation of recording electrodes at the superficial level of the cortex. The mice were anesthetized with isoflurane using a vaporizer system (Cristalia, 2.5%) during surgery and fixed to a mouse stereotaxic instrument (Kopf Instruments). Their heads were shaved and superficially opened at the top, the skullcap was cleaned, and then implanted with 1.5 mm-long gold-plated electrodes (stereotaxic coordinates: 1.0 mm length; 2 in the right and 2 in the left cortical surface).

To record the LFP signals, all mice were placed in a box measuring 41 cm × 34 cm × 18 cm containing clean sawdust. The receptor electrodes implanted were plugged with shielded cables, which were associated with a bio-signal amplifier (FE136 Animal Bio Amp – AD Instruments) and digitizer system (PowerLab 8/35 - AD Instruments), using LabChart 8 to display and save the data. All recordings were done 3 days after the surgery (12 dpi, the peak of viral replication in the brain). The procedures for the analysis of biological signs were approved by the Ethics and Research Committee of the Federal University of São Paulo, under the protocol number: 4495200219.

### Frequency bands characterization

The LFP records were initially filtered with notch to remove network noise, followed by division into 5-second windows with 75% overlap between them, to calculate the power spectra and the modulation index on phase and frequency. Recordings were first analyzed by their frequency band features, to investigate the spectral signature of the animals given their condition. Spectrograms were generated to evaluate power spectrum density (PSD) over time, through Fast Fourier Transform, and all frequency bands per animal were also calculated, using the same method. A confidence interval of the mean (CI, α = 5%) were calculated considering the Hamming windows as samples.

A principal component analysis (PCA) was performed to determine the clustering of brain rhythms between groups as well as the explained variation by each principal component. The following convention of brain rhythms was used: delta (1 ≤ δ < 4 Hz); theta (4 ≤ θ < 8 Hz); alpha (8 ≤ α < 12 Hz); beta 1 (12 ≤ β_1_ < 16 Hz); beta 2 (16 ≤ β_2_ < 30 Hz); and gamma (30 ≤ γ < 60 Hz)^50^.

### Empirical probability distributions

**Figure 6:**
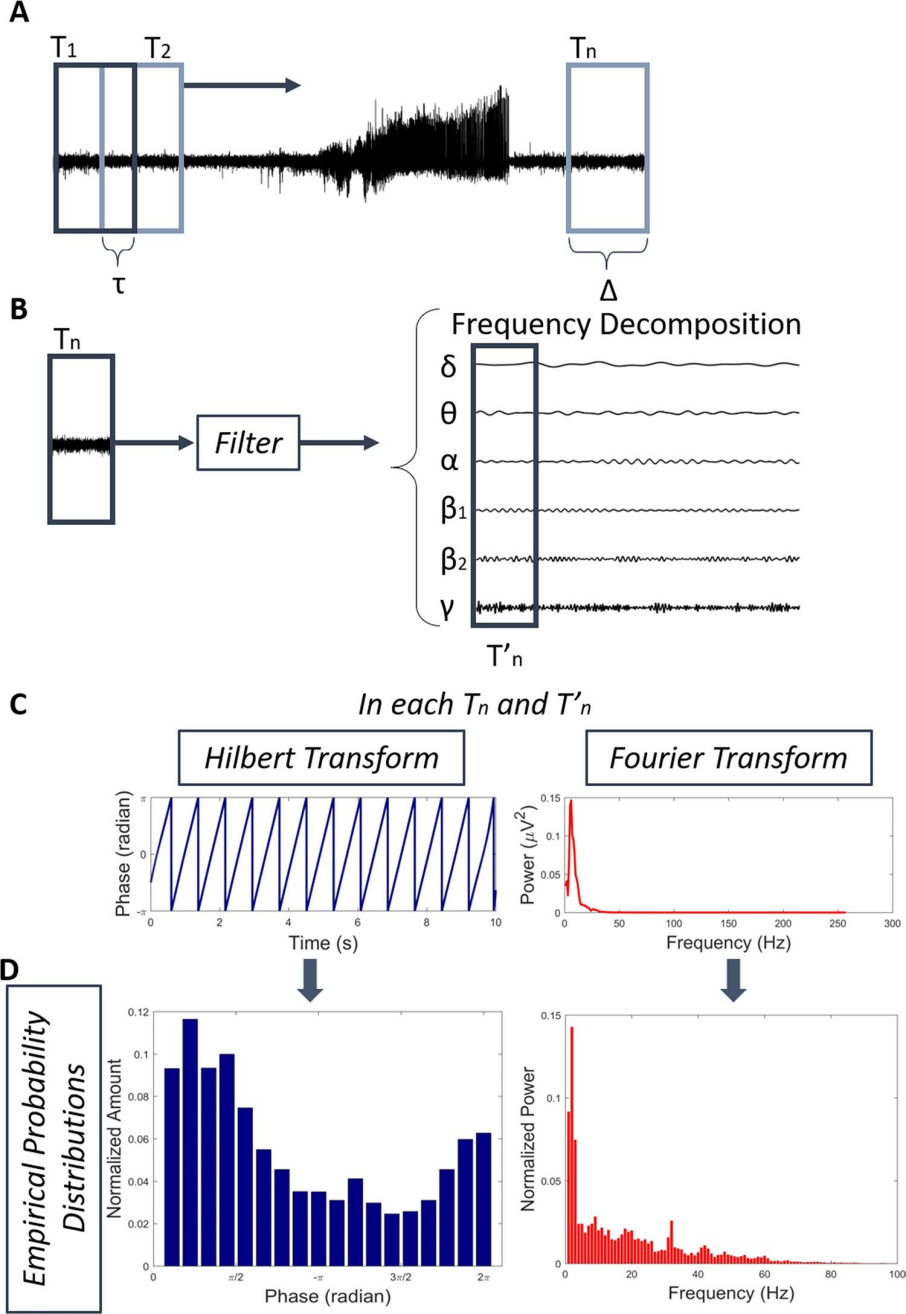
Methodology to calculate empirical probability distribution used to evaluate modulatory effects on phase and frequency. (**A**) The raw signal is segmented into stretches of same length (Δ) and with overlap of τ. (**B)** Each stretch is decomposed into six band frequency corresponding to the brain rhythms and then it is transformed to the frequency and phase using Fourier and Hilbert transform **(C)**. (**D)** It is calculated an empirical probability distribution from the results of the transforms used. These histograms are the basic unity of information used to calculate a ${\Lambda }_{j}^{k}$ index of modulatory effects, in which will be possible to analyze the modulation level of each rhythm.

To calculate the modulatory effects of signals from 8 mice (four by group), it was used the segments of 5 seconds, mentioned before, and then time-domain filtered in each of the brain rhythms, Fig. 6A,B. The time window sizes were heuristically calculated, optimizing the relationship between time and frequency features by minimizing the non-stationary variations per window using the Kolmogorov-Smirnov test to evaluate so. For each stretch of the specific filtered rhythm and the corresponding raw signal, empirical probability distributions were generated using frequency and phase features from the signal.

The paper proposes to identify the modulation levels of brain rhythms, using two general oscillatory features: (1) phase and (2) frequency, calculated from electrophysiological recordings *in vivo*. The empirical probability distributions are fundamental elements in analysis since it is the unity of information used to investigate the phenomenon of modulation in neural oscillations. In case of frequency modulations, the Power Spectrum Density (PSD) for each time window was calculated by using the module square power of the Fourier Transform. Then, each PSD was normalized by its sum up, in order to satisfy the two conditions of a probability distribution, namely, 0 < *p*(*i*) < 1 ∀ *i* and $\sum _{{\Omega }}\,p(i)=1$, in which Ω represents all the possible states.

For phase analysis, we used the Hilbert Transform to calculate their distribution in each time window. The distributions were calculated through histograms constructed by dividing the entire 360° phase-spectrum into eighteen classes of twenty phase degrees each^42^, and counting the number of events in each class. All phase histograms were then normalized independently, in order to satisfy the probability distribution conditions (Fig. 6D).

### Modulatory effects of phase and frequency

To evaluate the effects of modulation on each brain rhythm along the LFP records, we introduce the follow index:

1 $${\Lambda }_{j}^{k}(t)=\frac{{D}_{KL}({P}_{j}^{k}||{S}_{j})}{{D}_{KL}({P}_{j}^{k}||{U}_{j})}$$

wherein the index *j* refers to phase or frequency, respectively, *j* = {ph, fr}, and *k* = {δ, θ, α, β_1,_ β_2_ or γ}. The term ${P}_{j}^{k}$ is the empirical distribution of a given *j* and brain rhythm *k*, and S and U are respectively the raw signals and a uniform distribution. The uniform distribution, used in calculation of the index, is generated by dividing one by the number of states in each *j*, thus implying the same probability of occurrence of each state.

The temporal dependence of ${\Lambda }_{j}^{k}$ reinforces the fact that the modulatory effects must be analyzed continuously in order to evaluate all transitions along the entire signal. Even though the index has the same formula for both phase and frequency, its meaning and interpretation are not the same. This index uses the relative entropy or Kullback-Leibler divergence (D_*KL*_), which can be roughly interpreted as the asymmetrical cost of all possible states {*X*} from probability distribution $\tilde{P}$ to another $\tilde{Q}$ ^25^.

2 $${D}_{KL}(\tilde{P}||\tilde{Q})=\,-\,\sum _{x\,\in \,{\rm{X}}}\tilde{P}(x)\,\log \left(\frac{\tilde{Q}(x)}{\tilde{P}(x)}\right),$$

Considering phase, the index ${\Lambda }_{ph}^{k}$(*t*) demonstrates how the phase distribution of a rhythm *k* (δ, θ, α, β_1_, β_2,_ or γ) tends to a uniform-like distribution. A uniform-like phase distribution means that the sign has no coherent tendency; since, no specific phase regime has a greater probability of occurrence, this represents a state that is less selective to synchronization. Therefore, the lower the cost to transform a specific rhythm/phase distribution into a uniform-like distribution, the higher will be the probability to synchronize it with an oscillation pattern interacting with it. This means that the rhythm does not have a specific regime of synchronization and is not selective to interaction with other oscillations. Thus, the more similar the phase distribution of the rhythms, the more likely they are to synchronize. A uniform-like distribution the probability to synchronize with any rhythm (or oscillator) is higher, because it doesn’t have an specific phase regime of oscillation, and the phase differences with any oscillator will be the same, which by consequence does not stablish a preference for synchronization. The neural populations are coupled, they have trend to synchronize to the group of neurons with the lower phase difference.

Since D_KL_ (${P}_{j}^{k}$||${S}_{j}$) indicates how much a rhythm *j* explains the signal ${S}_{j}$, normalizing it by the divergence with uniform distribution, we consider coherent effects along the signal associated with specific rhythms.

In the frequency, the index ${\Lambda }_{fr}^{k}$(*t*) also amplifies the modulatory effects through their variation along time. Here, the uniform distribution on the denominator is needed to provide a comparison between evaluated records, since a uniform distribution corresponds to a state of maximum entropy^29^. Thus, D_KL_ (${P}_{j}^{k}$||${S}_{j}$) indicates how a specific spectral distribution ${P}_{j}^{k}$ is prevalent in the spectral distribution of the whole signal ${S}_{j}$. In this way, the ${\,\Lambda }_{fr}^{k}$(*t*) index describes how much information the spectral signature of a specific brain rhythm provides about the signal. Since any change in spectral frequency signature of a given recording corresponds to some modulatory effect, any change of the ${\Lambda }_{fr}^{k}$(*t*) along the time will indicate that there is some factor modulating the signal in that specific rhythm.

Figure 1 shows a general scheme of the signal processing steps used to calculate the proposed index ${\,\Lambda }_{j}^{k}$(*t*) for both phases and frequencies. Signals from ZIKV-infected mice were divided into four stages: (1) baseline activity, (2) preictal (five minutes of recording before seizure), (3) seizure (from generalized discharge to the suppressed electrical activity) and (4) postictal (five minutes of recording after the seizure), while the Mock-infected mice exhibited only one stage corresponding to baseline activity. All data were evaluated along these stages in order to determine different patterns associated with each brain rhythm that yields modulatory effects.

### Computational model: epileptors

We use the computational model of epilepsy, Epileptor^67^, in order to evaluate if the patterns found in the electrophysiological recordings were specifically related to ZIKV infection or to a common epileptic effect. All signals generated from Epileptors followed the parameters and equations described in the Supplementary Material. These parameters were chosen to simulate variations between fast and slow oscillations in an epileptiform activity.

### Statistical analysis

Shapiro-Wilk’s test was applied to verify normalcy. Representations for statistical comparisons were made using mean/median ± CI for parametric and non-parametric data, respectively. To verify possible differences between ${\,\Lambda }_{j}^{k}$(*t*) index associated with each recording stages (baseline, preictal, ictal, and postictal), the corresponding histograms were calculated and compared with respect to their four statistical moments: mean, standard deviation, skewness, and kurtosis (∀ *k* and *j*). A Kruskall-Wallis test were performed to analyze the differences between the modulation levels between the brain rhythms in phase and frequency. For all analyses, a significance level α = 5% was used, and all signal processing and statistical analyses were performed using Matlab^®^ 2017.

## Supplementary information

Supplementary Information.
