# Supplementary material for: Modulation in phase and frequency of neural oscillations during epileptiform activity induced by neonatal Zika virus infection in mice
Source: Sci Rep. 2020 Apr 21;10:6763. doi: 10.1038/s41598-020-63685-2 (PMC7174408; doi:10.1038/s41598-020-63685-2)
Supplement: Supplementary file 1 — Supplementary Information. [file 41598_2020_63685_MOESM1_ESM.docx]

**Modulation in phase and frequency of neural oscillations during epileptiform activity induced by neonatal Zika virus infection in mice**

Daniel J. L. L. Pinheiro^1*^, Leandro F. Oliveira^1^, Isis N. O. Souza^2^, João A. Ferres Brogin^3^, Douglas D. Bueno^4^, Iranaia Assunção Miranda^5^, Andrea T. Da Poian^6^, Sergio T. Ferreira^6,7^, Claudia P. Figueiredo^2^, Julia R. Clarke^2^; Esper A. Cavalheiro^1^; Jean Faber^1,8^

^1^ Department of Neurology and Neurosurgery – Paulista School of Medicine – Federal University of São Paulo (UNIFESP).

^2^ School of Pharmacy – Federal University of Rio de Janeiro (UFRJ), Rio de Janeiro, RJ, 21944-590, Brazil.

^3^ Department of Mechanical Engineering – São Paulo State University, Ilha Solteira, SP, 15385-000, Brazil.

^4^ Department of Mathematics – São Paulo State University, Ilha Solteira, SP, 15385-000, Brazil.

^5^ Institute of Microbiology Paulo de Goes, Federal University of Rio de Janeiro (UFRJ), Rio de Janeiro, RJ, 21944-590, Brazil

^6^ Institute of Medical Biochemistry Leopoldo de Meis, Federal University of Rio de Janeiro (UFRJ), Rio de Janeiro, RJ, 21944-590, Brazil.

^7^ Institute of Biophysics Carlos Chagas Filho, Federal University of Rio de Janeiro (UFRJ), Rio de Janeiro, RJ, 21944-590, Brazil.

^8^ Nucleus of Neuroengineering and Computation – Institute of Science and Technology – Federal University of São Paulo (UNIFESP).

* Corresponding author:

Daniel José Lins Leal Pinheiro

Department of Neurology and Neurosurgery – Paulista School of Medicine – Federal University of São Paulo (UNIFESP)

669, Pedro de Toledo St., 2nd floor, Vila Clementino, São Paulo, SP, Brazil. Zip code: 04039-032.

Tel: +55 (11) 5576-4848 - Voip: 2838

E-mail: pdanielleal@gmail.com

[**Supplementary information**](https://static-content.springer.com/esm/art%3A10.1038%2Fs41598-019-40969-w/MediaObjects/41598_2019_40969_MOESM1_ESM.docx)


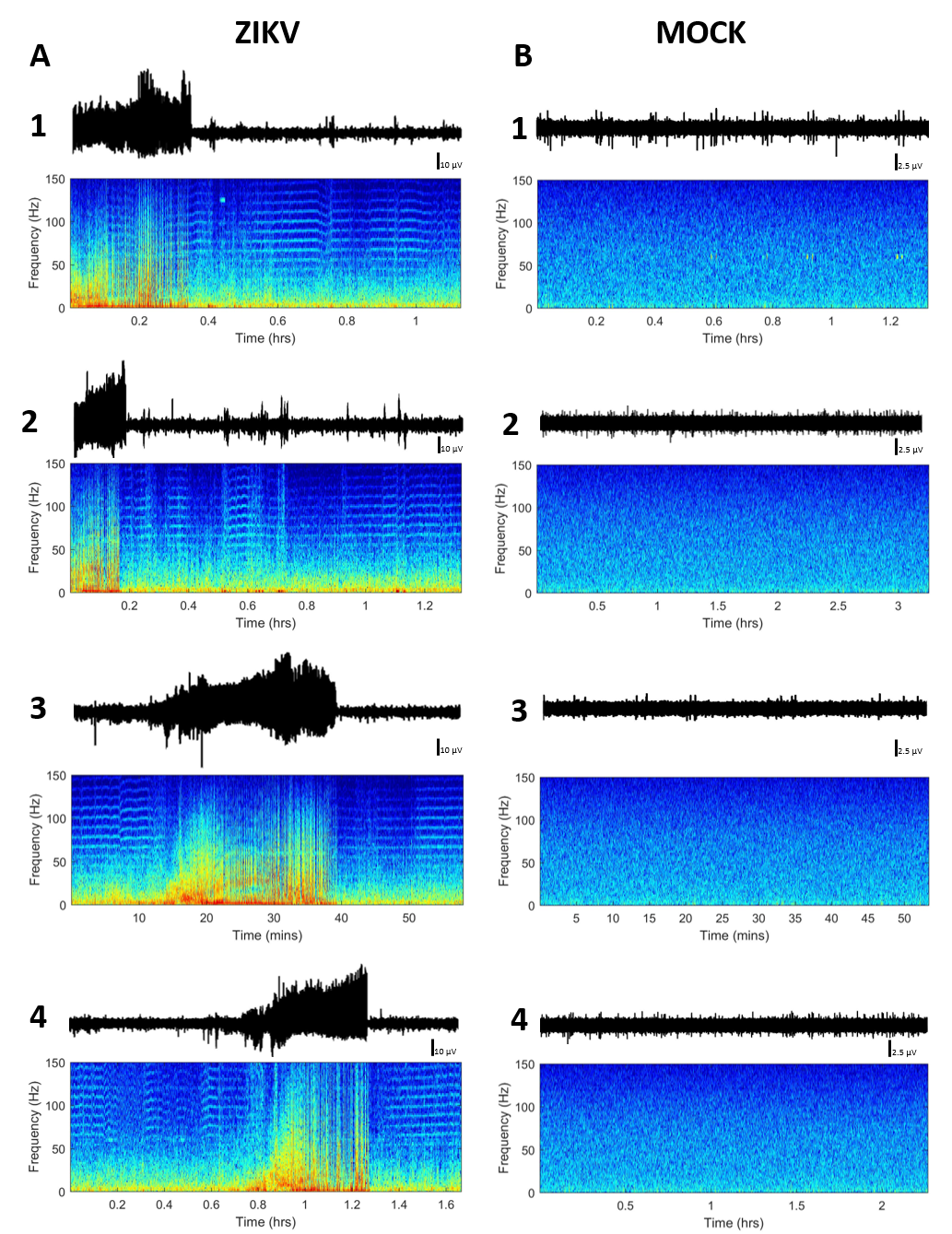


**Figure S1:** **Electroencephalographic recording and spectrogram of the activities of all neonate mice.** (A) Animals in the ZIKV-infected group and (B) Animals in the control group (Mock). In (A) there is a spectral signature that privileges certain low-frequency bands, even outside the epileptiform activity, while at the beginning of the crisis there is a predominance of bands related to rapid oscillations. The same effect is not observed in the animals of the Mock group, which do not emphasize the prevalence of any specific frequency band.

**Computational Model: Epileptors**

The Epileptor model is a phenomenological canonical model which can reproduce the most common features that are present in epileptic-like activities. Considering the hypothesis that some of these features are invariant across species, Jirsa and colleagues, after analyzing signals obtained from different species (human, mouse, rat and zebrafish) to verify this premise and carrying out a thorough statistical analysis, proposed the first version of Epileptor, which is based on an experimental model considering local field potentials (LFPs) from the hippocampus of mice, and is designed considering the aid of nonlinear dynamics tools (JIRSA et al., 2014). The same authors also proposed a taxonomy of seizure-like events according to the bifurcations verified in such signals.

Although seven different patterns can be identified after seizures onset, there are two predominant ones (JIRSA et al., 2014): fast oscillations and spike (or spike and wave) events, which are often, but not necessarily, coupled. An illustration of such behaviors can be found in Figure 1, whose curves were obtained using simulations of Epileptor considering 2 cases: without superposition of the oscillators (Fig. 1a) (that is, the solutions are plotted separately), and with superposition of the oscillators (Fig. 1b), both given in terms of local field potentials (LFP).

**Figure S2:** a) to the left, both epileptic-like activities (fast oscillations and spike and wave events) with no superposition between the curves; b) to the right, the same curves, but after superposing them.

According to the experiments carried out by Jirsa and colleagues (JIRSA et al., 2014), the predominant dynamic structures present in the experimental model, which then led to Epileptor, are: i) the inter-spike intervals follow a logarithmic scale as the system approaches seizure offset; ii) seizure onset usually defines an abrupt baseline shift, thus characterizing a saddle-node bifurcation; iii) seizure offset is characterized by another shift back up to the baseline, which (along with the inter-spike logarithmic scale) represents the second bifurcation, known as homoclinic. A more detailed review on bifurcations and neural dynamics can be found in Izhikevich, 2000. To represent this behavior as a mathematical model, a set of first-order differential equations was proposed, comprising individual sub-systems associated to the seizure-related activities mentioned before:

$$\left\{ \begin{aligned} \dot{x}_{1}=y_{1}-f_{1}\left( x_{1},y_{1} \right)-z+I_{rest1} \\ \dot{x}_{1}=y_{0}-5x_{1}^{2}-y_{1} \\ \begin{matrix} \dot{z}=\frac{1}{\tau_{0}}[4\left( x_{1}-x_{0} \right)-z] \\ \begin{matrix} \dot{x}_{2}=-y_{2}+x_{2}-x_{2}^{3}+I_{rest2}+0.002g\left( x_{1} \right)-0.3(z-3.5) \\ \dot{y}_{2}=\frac{1}{\tau_{2}}[-y_{2}+f_{2}(x_{2})] \end{matrix} \end{matrix} \end{aligned} \right.$$

where:

$$f_{1}(x_{1},x_{2})\left\{ \begin{matrix} x_{1}^{3}-3x_{1}^{2}, if x_{1}<0 \\ \left[ x_{2}-0.6\left( z-4 \right)^{2} \right]x_{1}, if x_{1}\geq0 \end{matrix} \right.$$

$$f_{2}(x_{2})\left\{ \begin{matrix} 0, if x_{2}<-0.25 \\ 6\left( x_{2}+0.25 \right), if x_{2}\geq-0.25 \end{matrix} \right.$$

$$g\left( x_{1} \right)=\int_{t_{0}}^{t} e^{-\gamma\left( t-\tau\right)}x_{1}(\tau)d\tau$$

The equation for $\dot{x}_{2}$ can be rewritten, as defined by Jirsa et al. (2014) and highlighted by Nagaraj et al. (2017), in a more convenient form for numerical integration purposes considering the following change of variables:

$$\dot{x}_{2}=-y_{2}+x_{2}-x_{2}^{3}+I_{rest2}+2u(t)-0.3(z-3.5)$$

where:

$$u\left( t \right)=-\gamma(u-x_{1})$$

A more detailed description about the meaning of all the variables can be found in Jirsa et al. 2014. However, in terms of electrical activity, two main variables compose the local field potentials: $x_{1}$ and $x_{2}$ (more precisely: $-x_{1}+x_{2}$ ). The first one is linked to fast oscillations (and, along with $y_{1}$, compose the first subsystem); the second one is related to the spike and wave events (and, along with $y_{2}$, compose the second subsystem); variable $z(t)$ is known as permittivity, and is a control variable, whose behavior over time may drive the system into and out of seizures, depending whether extreme values are reached; variables $I_{rest1}$ and $I_{rest2}$ are associated to the flow of electric currents moving into or out of the neural cells; $\tau$ represents the time scale control parameter, that is, it is linked to how fast the trajectories on the phase portraits (and, consequently, in time) evolve (the following relation holds for Epileptor: $\tau_{1}$ < $\tau_{2}$ < $\tau_{0}$); $x_{0}$ is known as the degree of epileptogenicity, that is, depending on its value, Epileptor is placed in a persistent epileptic condition, presenting variable seizure-like events in terms of intensity and duration; $f_{1}(x_{1},x_{2})$ and $f_{2}\left( x_{2} \right)$, in turn, are coupling functions, which allow both subsystems to synchronize over time, and were designed based on the fact that they are often coupled (JIRSA et al., 2014); $y_{0}$ and $\gamma$, at last, are constant parameters associated to the model: the first one is defined by Hindmarsh and Rose (1984); the second one, in turn, is added by Jirsa et. al (2014).

**Adapted Epileptor’s dynamics:**

Since Epileptor’s inherent dynamics, despite presenting important features of epileptic seizures, does not exactly reproduce other aspects observed in the real signals obtained in this work (such as the transition to the ictal state centered on the baseline, or the increase in the amplitude of spikes over time, for example), an attempt to adapt its mathematical model so that an approximate behavior is attained was carried out. The modifications made in the original model are as follows:

$$\left\{ \begin{aligned} \dot{x}_{1}=\frac{1}{\tau_{1}}[y_{1}-f_{1}\left( x_{1},y_{1} \right)-z+I_{rest1}] \\ \dot{y}_{1}=\frac{a}{\tau_{1}}(y_{0}-bx_{1}^{2}-y_{1}) \\ \begin{matrix} \dot{z}=\frac{1}{\tau_{0}}[4\left( x_{1}-x_{0} \right)-z] \\ \begin{matrix} \dot{x}_{2}=c[-f_{3}\left( t \right)y_{2}+x_{2}-x_{2}^{3}+I_{rest2}+0.002g\left( x_{1} \right)-0.15\left( z-3.5 \right)] \\ \dot{y}_{2}=\frac{1}{\tau_{2}}[-y_{2}+f_{2}(x_{2})] \end{matrix} \end{matrix} \end{aligned} \right.$$

where:

$$f_{1}(x_{1},x_{2})\left\{ \begin{matrix} x_{1}^{3}-3x_{1}^{2}, if x_{1}<0 \\ \frac{1}{h}(dx_{1}x_{2}-ex_{1}z), if x_{1}\geq0 \end{matrix} \right.$$

$$f_{2}(x_{2})\left\{ \begin{matrix} 0, if x_{2}<-0.25 \\ 6\left( x_{2}+0.25 \right), if x_{2}\geq-0.25 \end{matrix} \right.$$

$$g\left( x_{1} \right)=\int_{t_{0}}^{t} e^{-\gamma\left( t-\tau\right)}x_{1}(\tau)d\tau$$

where $\tau_{1}=0.3$, $\tau_{2}=2$, $\tau_{0}=2857$, $I_{rest1}=3.2$, $I_{rest2}=0.45$, $\gamma=0.01$, $x_{0}=-1.6$, $y_{0}=1$, $a=2$, $b=5.5$, $c=6.67$, $d=0.95$, $e=0.21$ and $h=0.9$. It must be stressed that the same equation for transforming $g\left( x_{1} \right)$ into $u\left( t \right)$ must be considered. In particular, $d$ defines the coupling strength between $x_{1}$ and $x_{2}$ in $f_{1}(x_{1},x_{2})$, and $e$, in turn, the coupling strength between $x_{1}$ and $z$ in $f_{1}(x_{1},x_{2})$. Note that, for the latter one, such a coupling is not a function given by a quadratic term of $z$ anymore. These modifications are intended to keep the fast oscillations at a relatively more constant amplitude over time, as seen in the signals from mice with Zika virus. $f_{3}\left( t \right)$ is a simple linear function of time, whose initial value was fixed at a positive constant when the simulations approached the beginning of a seizure and, after each iteration, it decreased its value by a pre-defined step. This procedure is intended, thereby, to cause the spike and wave events to increase in amplitude over time.

At last, the superposition that defines the local field potentials (${-x}_{1}+x_{2}$) was adapted to: ${s_{1}x}_{1}+{s_{2}x}_{2}$, where $s_{1}$ and $s_{2}$ are also constants. Given that the transition to the ictal state is centered on the baseline (and, thus, does not present a shift up or down from it, as originally designed for Epileptor), this modification was added to try to circumvent this difference in behavior. The values that $s_{1}$ and $s_{2}$ assume depend on how expressive the two main behaviors (fast oscillations and spike and wave events) are intended to have. In our case, $s_{1}<s_{2}$, not to minimize the fast oscillations of $x_{1}$ themselves, but rather to minimize the shifting effect. It must be stressed that, for the purposes of this work, deeper modifications in the model (considering a thorough nonlinear analysis, involving bifurcations, for example) are not carried out.

For the sake of understanding, Figure S3 presents a comparison considering three cases: Epileptor’s original dynamics, Epileptor’s adapted dynamics, and a real signal; also, Figure S4 shows enlarged views of the adapted Epileptor and the same signal. The simulations were implemented using a fourth-order Runge-Kutta integrator, and the parameters adopted for the original Epileptor are: $x_{0}=-1.6$, $y_{0}=1$,$\tau_{0}=2857$, $\tau_{1}=1$, $\tau_{2}=10$, $I_{rest1}=3.1$, $I_{rest2}=0.45, \gamma=0.01$, as defined by Jirsa et al. (2014). The initial conditions for both original and adapted model are the same: $x_{1}\left( 0 \right)=0$, $y_{1}\left( 0 \right)=-5$, $z\left( 0 \right)=3$ $x_{2}\left( 0 \right)=0$, $y_{2}\left( 0 \right)=0$ and $u\left( 0 \right)=0$. For the two simulated signals, at each iteration a sample from a gaussian distribution with zero mean and unity variance, multiplied by a scaling factor was added to $y_{2}$ to introduce a more stochastic behavior to the system. This procedure was not carried out for $x_{1}$ or $y_{1}$ to better preserve the fast oscillation structures. Also, noise was added to the output, that is, a gaussian distribution (zero mean and unity variance) of the same length as variables $x_{1}$ and $x_{2}$, multiplied by a scaling factor and the Root Mean Square value (RMS) of the LFP, as a superposition to the simulated signal, to account for possible noise in the output (measurement noise). Note that the adapted dynamics better represents the increase in amplitude, the sudden seizure offset, and a more centered transition from the two states compared to the real signal, while keeping the two main features present, fast oscillations and spike and wave events, whereas Epileptor’s original dynamics only accounts for the two main features (see its enlarged view in Fig. S2).

**Figure S3:** Comparison between the behaviors of the original Epileptor model (top), the adapted Epileptor model (middle), and a real signal (bottom).

As verified from the simulations and real signals, both present the main epileptic activities: fast oscillations and spike and wave events, which also seem to be coupled to each other (as shown in Fig. S2). This suggests the existence of activities whose attractors are similar to those observed in Jirsa et al. (2014). Furthermore, the baseline condition and epileptic seizures are well defined events, represented by transitions in terms of behavior, which may indicate that bifurcations are, indeed, part of this type of seizure. In this point, the model represents well such features. Since Epileptor presents seizure onsets as baseline shifts to high-oscillating activities, as well as seizure offsets as shifts from the epileptic activity back to the baseline condition, and considering that such a characteristic was not verified in our experimental signals, this suggests that different kinds of bifurcations may define their behavior.

**Figure S4:** Enlarged view of both adapted model (top) and real signal (bottom).

Considering the similarities and differences observed between the mathematical model and the real signals, it is possible to state that Epileptor represents the epileptic seizures obtained from the mice used in the experiments of this work fairly well, accounting for the spike and wave events, fast oscillations, and transitions in terms of behavior: from the baseline to the epileptic seizure, and from the seizure back to the baseline condition. Nevertheless, from a theoretical point of view, even if some of main features are correctly captured by Epileptor, it may not represent the bifurcations existing in the real signals. It must be stressed that the experimental model used by Jirsa et al. (2014) was based on mice that did not present the Zika virus (full details concerning the experimental procedures can be found in the same paper). Therefore, this suggests that these epileptic activities might have their own inherent dynamics, which remains unclear and needs to be addressed in further work. A more detailed, comprehensive study about the nonlinear features present in the signals acquired (such as a bifurcation analysis, for example) may help find evidence for whether they are directly connected to the Zika virus or not.

At last, due to the fact the $s_{1}<s_{2}$, the fast oscillations present a reduced amplitude in the adapted Epileptor model, whereas they are at a similar level as the spikes in the real signals (Fig. 6); conversely, even if the original Epileptor model presents fast oscillations with more realistic amplitudes, the other relevant features are less pronounced or not present. Therefore, even being able to approximate the desired behaviors and providing helpful insights related to which parameters and functions to adjust for doing so, the adapted Epileptor model needs further work, considering a more detailed nonlinear analysis and suitable bifurcations, to account for both features at an appropriate level.

Note that the adapted dynamics better represents the increase in amplitude, the sudden seizure offset, and a more centered transition from the two states compared to the real signal, while keeping the two main features present: fast oscillations and spike and wave events; whereas Epileptor’s original dynamics only accounts for the two main features. The figure S5, represents an example of the two types of signals generated by the model of Epileptors as well the results obtained by the application of $\Lambda_{j}^{k}$.


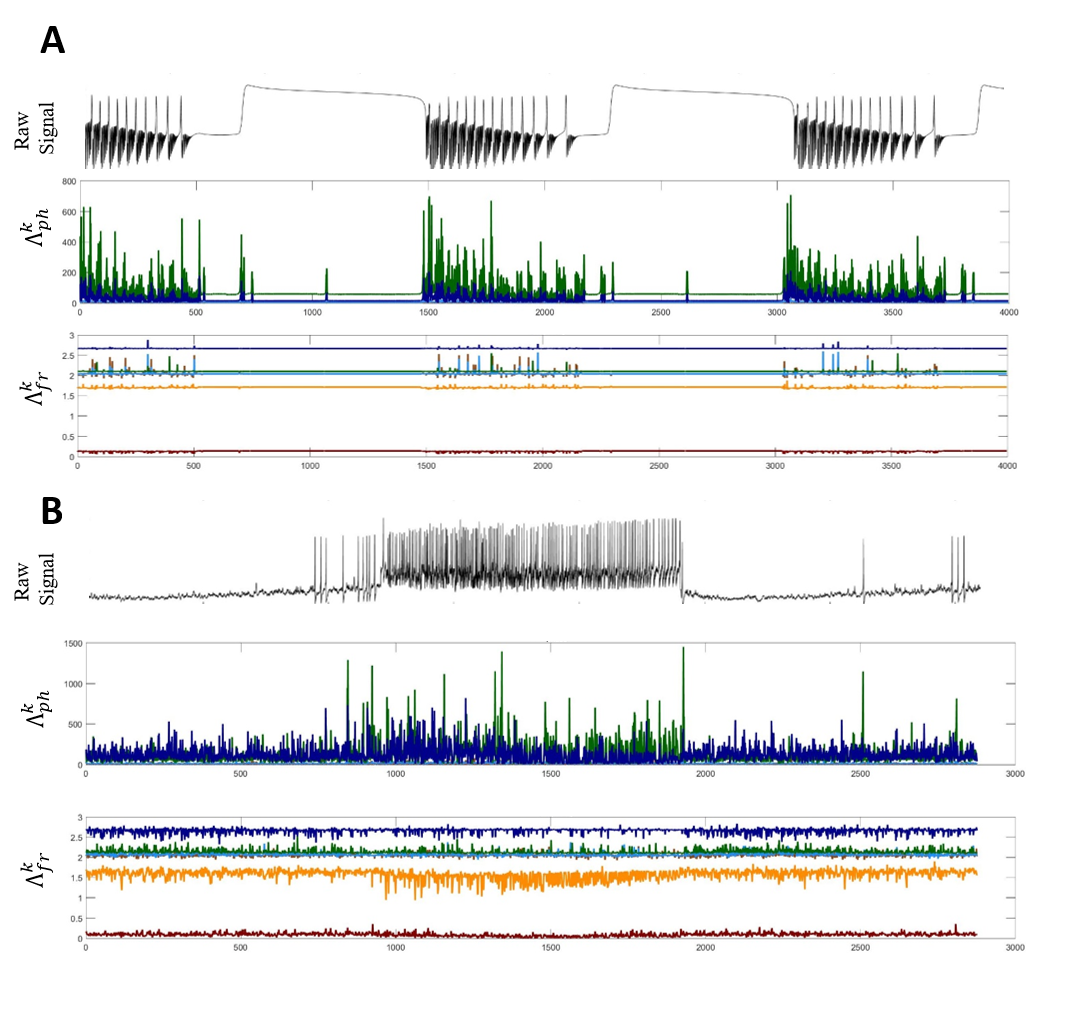


**Figure S5: Analysis of $\boldsymbol{\Lambda}_{\boldsymbol{j}}^{\boldsymbol{k}}$ on signals generated from Epileptor computational model.** A) Signal using the original parameters of the model and its respective phase and frequency modulation given by $\boldsymbol{\Lambda}_{\boldsymbol{j}}^{\boldsymbol{k}}$. B) Signal using adapted parameters to include a basal activity before the epileptic seizure and its respective phase and frequency modulation given by $\boldsymbol{\Lambda}_{\boldsymbol{j}}^{\boldsymbol{k}}$. It is possible to observe a similar pattern of modulation on frequency and phase as found in the electrophysiological activities from animals infected with ZIKV. Both Epileptors signals seems to be a good alternative to compare and evaluate proper differences associated to different mechanisms of epileptiform activities.

All the implementations are available in: <https://github.com/lheureduloup/epileptor>.

**References:**

HINDMARSH, J. L.; ROSE, R. M.; HUXLEY, A. F. A model of neuronal bursting using three coupled first order differential equations. Proceedings of the Royal Society of London. Series B. Biological Sciences, v. 221, n. 1222, p. 87--102, 1984. Available from: @https://royalsocietypublishing.org/doi/abs/10.1098/rspb.1984.0024A.

IZHIKEVICH, E. M. Neural excitability, spiking and bursting. International Journal of Bifurcation and Chaos, v. 10, n. 06, p. 1171--1266, 2000. Available from: @https://doi.org/10.1142/S0218127400000840A.

JIRSA, V. K.; STACEY, W. C.; QUILICHINI, P. P.; IVANOV, A. I.; BERNARD, C. On the nature of seizure dynamics. Brain, v. 137, n. 8, p. 2210--2230, 06 2014. ISSN 0006-8950. Available from: @https://doi.org/10.1093/brain/awu133A.

NAGARAJ, V.; LAMPERSKI, A.; NETOFF, T. I. Seizure control in a computational model using a reinforcement learning stimulation paradigm. International journal of neural systems, v. 27, n. 07, p. 1750012, 2017.

**Statistical comparisons of Brain Rhythms per stage for modulation on phase**

The following tables refer to the results obtained with the Kruskal-Walis test to verify the significant differences for the levels of modulation of brain rhythms in the phase between the different signal stages and experimental group.

**Table S1:** p-Values from comparisons between Delta Rhythm modulation on phase

|  | **Baseline Mock** | **Baseline ZikV** | **Preictal ZikV** | **Seizure ZikV** | **Postictal ZikV** |
| --- | --- | --- | --- | --- | --- |
| **Baseline Mock** | - | 0.5702 | 0.1573 | 0.0016 | 0.8390 |
| **Baseline ZikV** | - | - | 1.0000 | 1.0000 | 1.0000 |
| **Preictal ZikV** | - | - | - | 0.8390 | 0.9772 |
| **Seizure ZikV** | - | - | - | - | 0.1573 |
| **Postictal ZikV** | - | - | - | - | - |

**Table S2:** p-Values from comparisons between Theta Rhythm modulation on phase

|  | **Baseline Mock** | **Baseline ZikV** | **Preictal ZikV** | **Seizure ZikV** | **Postictal ZikV** |
| --- | --- | --- | --- | --- | --- |
| **Baseline Mock** | - | 0.8062 | 0.1573 | 0.0016 | 0.6603 |
| **Baseline ZikV** | - | - | 0.9969 | 0.3342 | 1.0000 |
| **Preictal ZikV** | - | - | - | 0.8390 | 0.9975 |
| **Seizure ZikV** | - | - | - | - | 0.2826 |
| **Postictal ZikV** | - | - | - | - | - |

**Table S3:** p-Values from comparisons between Alpha Rhythm modulation on phase

|  | **Baseline Mock** | **Baseline ZikV** | **Preictal ZikV** | **Seizure ZikV** | **Postictal ZikV** |
| --- | --- | --- | --- | --- | --- |
| **Baseline Mock** | - | 0.7528 | 0.1837 | 0.0016 | 0.6603 |
| **Baseline ZikV** | - | - | 0.9994 | 0.3883 | 1.0000 |
| **Preictal ZikV** | - | - | - | 0.7995 | 0.9988 |
| **Seizure ZikV** | - | - | - | - | 0.2826 |
| **Postictal ZikV** | - | - | - | - | - |

**Table S4:** p-Values from comparisons between Beta 1 Rhythm modulation on phase

|  | **Baseline Mock** | **Baseline ZikV** | **Preictal ZikV** | **Seizure ZikV** | **Postictal ZikV** |
| --- | --- | --- | --- | --- | --- |
| **Baseline Mock** | - | 0.6946 | 0.2133 | 0.0016 | 0.6603 |
| **Baseline ZikV** | - | - | 0.9999 | 0.4464 | 1.0000 |
| **Preictal ZikV** | - | - | - | 0.7561 | 0.9995 |
| **Seizure ZikV** | - | - | - | - | 0.2826 |
| **Postictal ZikV** | - | - | - | - | - |

**Table S5:** p-Values from comparisons between Beta 2 Rhythm modulation on phase

|  | **Baseline Mock** | **Baseline ZikV** | **Preictal ZikV** | **Seizure ZikV** | **Postictal ZikV** |
| --- | --- | --- | --- | --- | --- |
| **Baseline Mock** | - | 0.7528 | 0.1837 | 0.0016 | 0.6603 |
| **Baseline ZikV** | - | - | 0.7995 | 0.03205 | 0.9994 |
| **Preictal ZikV** | - | - | - | 0.02527 | 0.3883 |
| **Seizure ZikV** | - | - | - | - | 2826 |
| **Postictal ZikV** | - | - | - | - | - |

**Table S6:** p-Values from comparisons between Gamma Rhythm modulation on phase

|  | **Baseline Mock** | **Baseline ZikV** | **Preictal ZikV** | **Seizure ZikV** | **Postictal ZikV** |
| --- | --- | --- | --- | --- | --- |
| **Baseline Mock** | - | 0.7528 | 0.2133 | 0.0016 | 0.6098 |
| **Baseline ZikV** | - | - | 0.9997 | 0.0134 | 1.0000 |
| **Preictal ZikV** | - | - | - | 0.0095 | 0.9998 |
| **Seizure ZikV** | - | - | - | - | 0.3222 |
| **Postictal ZikV** | - | - | - | - | - |

**Statistical comparisons of Brain Rhythms per stage for modulation on frequency**

The following tables refer to the results obtained with the Kruskal-Walis test to verify the significant differences for the levels of modulation of brain rhythms in the frequency between the different signal stages and experimental group.

**Table S7:** p-Values from comparisons between Delta Rhythm modulation on frequency

|  | **Baseline Mock** | **Baseline ZikV** | **Preictal ZikV** | **Seizure ZikV** | **Postictal ZikV** |
| --- | --- | --- | --- | --- | --- |
| **Baseline Mock** | - | 0.0325 | 0.3222 | 0.0206 | 0.9944 |
| **Baseline ZikV** | - | - | 0.8842 | 1.0000 | 0.9999 |
| **Preictal ZikV** | - | - | - | 0.9999 | 0.9772 |
| **Seizure ZikV** | - | - | - | - | 0.7561 |
| **Postictal ZikV** | - | - | - | - | - |

**Table S8:** p-Values from comparisons between Theta Rhythm modulation on frequency

|  | **Baseline Mock** | **Baseline ZikV** | **Preictal ZikV** | **Seizure ZikV** | **Postictal ZikV** |
| --- | --- | --- | --- | --- | --- |
| **Baseline Mock** | - | 0.0562 | 0.2463 | 0.7561 | 0.0460 |
| **Baseline ZikV** | - | - | 0.9848 | 0.6943 | 1.0000 |
| **Preictal ZikV** | - | - | - | 0.9988 | 0.9995 |
| **Seizure ZikV** | - | - | - | - | 0.8739 |
| **Postictal ZikV** | - | - | - | - | - |

**Table S9:** p-Values from comparisons between Alpha Rhythm modulation on frequency

|  | **Baseline Mock** | **Baseline ZikV** | **Preictal ZikV** | **Seizure ZikV** | **Postictal ZikV** |
| --- | --- | --- | --- | --- | --- |
| **Baseline Mock** | - | 0.1112 | 0.0135 | 0.7561 | 0.2463 |
| **Baseline ZikV** | - | - | 1.0000 | 0.9513 | 0.9999 |
| **Preictal ZikV** | - | - | - | 0..6098 | 0.9772 |
| **Seizure ZikV** | - | - | - | - | 0.9988 |
| **Postictal ZikV** | - | - | - | - | - |

**Table S10:** p-Values from comparisons between Beta 1 Rhythm modulation on frequency

|  | **Baseline Mock** | **Baseline ZikV** | **Preictal ZikV** | **Seizure ZikV** | **Postictal ZikV** |
| --- | --- | --- | --- | --- | --- |
| **Baseline Mock** | - | 0.2401 | 0.0167 | 0.4582 | 0.2826 |
| **Baseline ZikV** | - | - | 0.9987 | 1.0000 | 1.0000 |
| **Preictal ZikV** | - | - | - | 0.900 | 0.9772 |
| **Seizure ZikV** | - | - | - | - | 1.0000 |
| **Postictal ZikV** | - | - | - | - | - |

**Table S11:** p-Values from comparisons between Beta 2 Rhythm modulation on frequency

|  | **Baseline Mock** | **Baseline ZikV** | **Preictal ZikV** | **Seizure ZikV** | **Postictal ZikV** |
| --- | --- | --- | --- | --- | --- |
| **Baseline Mock** | - | 0.5075 | 0.0379 | 0.0135 | 0.8390 |
| **Baseline ZikV** | - | - | 0.9933 | 0.9456 | 0.9999 |
| **Preictal ZikV** | - | - | - | 1.0000 | 0.7561 |
| **Seizure ZikV** | - | - | - | - | 0.5079 |
| **Postictal ZikV** | - | - | - | - | - |

**Table S12:** p-Values from comparisons between Gamma Rhythm modulation on frequency

|  | **Baseline Mock** | **Baseline ZikV** | **Preictal ZikV** | **Seizure ZikV** | **Postictal ZikV** |
| --- | --- | --- | --- | --- | --- |
| **Baseline Mock** | - | 0.5075 | 0.2826 | 0.0108 | 0.3222 |
| **Baseline ZikV** | - | - | 1.0000 | 0.9261 | 1.0000 |
| **Preictal ZikV** | - | - | - | 0.9495 | 1.0000 |
| **Seizure ZikV** | - | - | - | - | 0.9292 |
| **Postictal ZikV** | - | - | - | - | - |
